# Correction: ATRIP protects progenitor cells against DNA damage in vivo

**DOI:** 10.1038/s41419-020-03227-w

**Published:** 2020-12-15

**Authors:** Gabriel E. Matos-Rodrigues, Paulius Grigaravicius, Bernard S. Lopez, Thomas G. Hofmann, Pierre-Olivier Frappart, Rodrigo A. P. Martins

**Affiliations:** 1 Programa de Biologia Celular e do Desenvolvimento, Instituto de Ciências Biomédicas, Universidade Federal do Rio de Janeiro, Rio de Janeiro, Brazil; 2 Clinical Cooperation Unit Neuropathology, German Cancer Research Center (DKFZ), D-69120 Heidelberg, Germany; 3 Institut Cochin, INSERM U1016, UMR8104 CNRS, Université Paris-Descartes, Equipe Labellisée Ligue Contre le Cancer, 24 rue du Faubourg St Jacques, 75014 Paris, France; 4 Institute of Toxicology, University Medical Center of the Johannes Gutenberg University Mainz, Mainz, Germany; 5 Present Address: Institut Cochin, INSERM U1016, UMR8104 CNRS, Université Paris-Descartes, Equipe Labellisée Ligue Contre le Cancer, 24 rue du Faubourg St Jacques, 75014 Paris, France; 6 Present Address: Leibniz Institute on Aging - Fritz Lipmann Institute (FLI), D-07745 Jena, Germany

**Keywords:** Checkpoints, Cell proliferation, Disease model, DNA replication, DNA damage response

Correction to: *Cell Death and Disease*

10.1038/s41419-020-03090-9 published online 28 October 2020

The original version of this Article contained an error in the name of author Thomas G. Hofmann, which was incorrectly given as Thomas Hofmann.

This has now been corrected in both the PDF and HTML versions of the Article.

