# Supplementary figures and images for: ATRIP protects progenitor cells against DNA damage in vivo
Source: Cell Death Dis. 2020 Oct 28;11(10):923. doi: 10.1038/s41419-020-03090-9 (PMC7591577; doi:10.1038/s41419-020-03090-9)

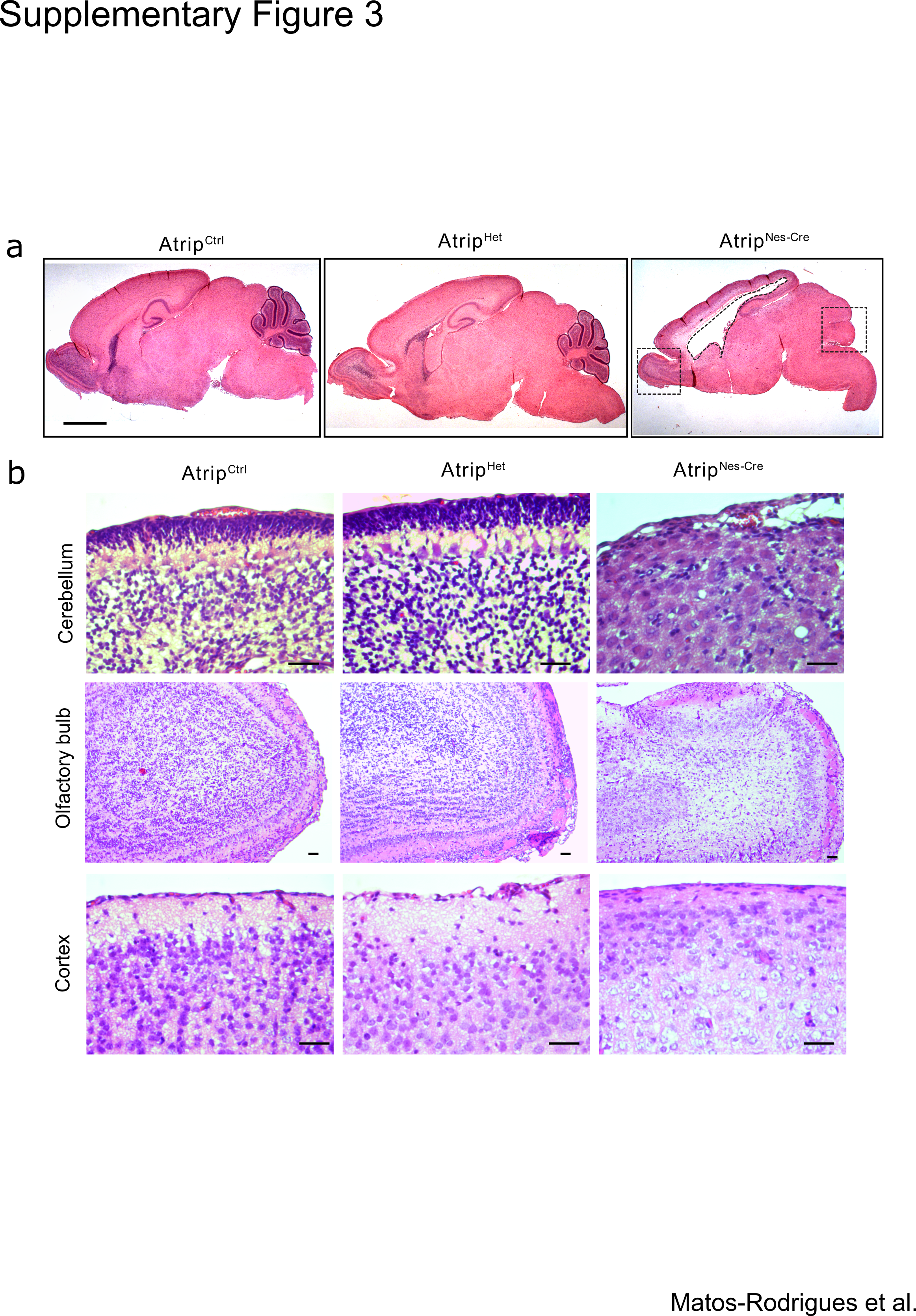

Supplement: Supplementary file 1 — SUPP FIG 1 [file 41419_2020_3090_MOESM1_ESM.png]

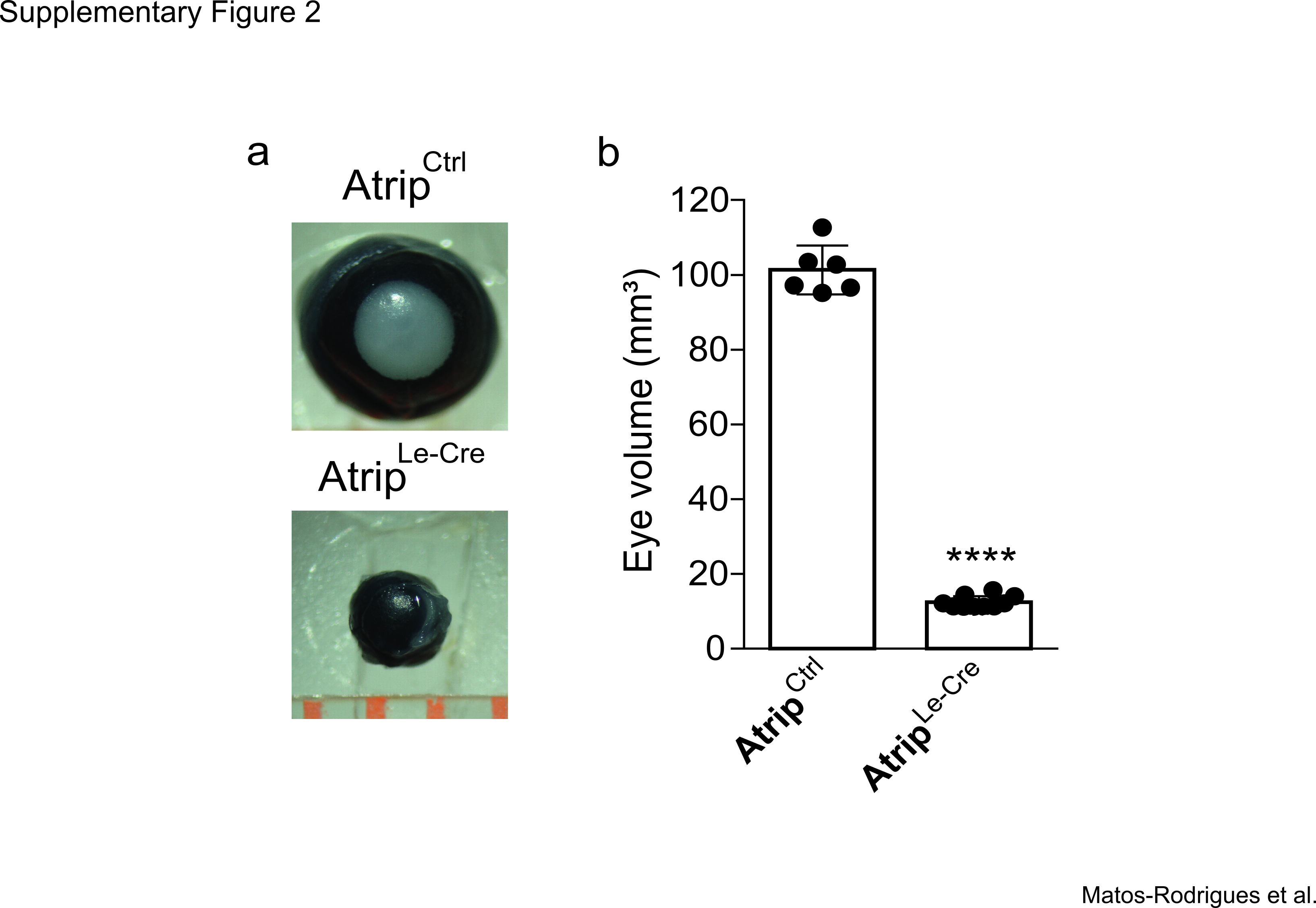

Supplement: Supplementary file 2 — SUPP FIG 2 [file 41419_2020_3090_MOESM2_ESM.png]

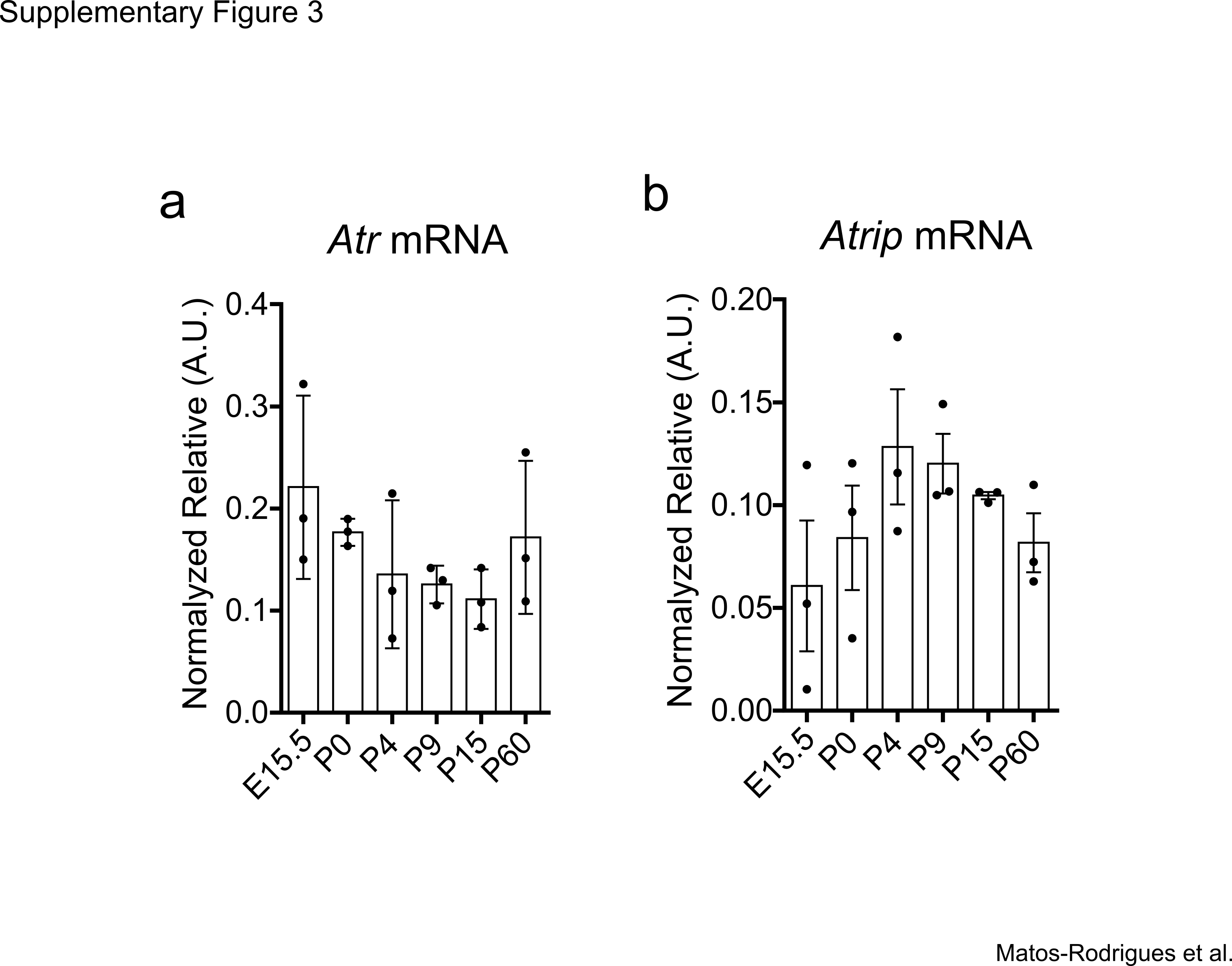

Supplement: Supplementary file 3 — SUPP FIG 3 [file 41419_2020_3090_MOESM3_ESM.png]

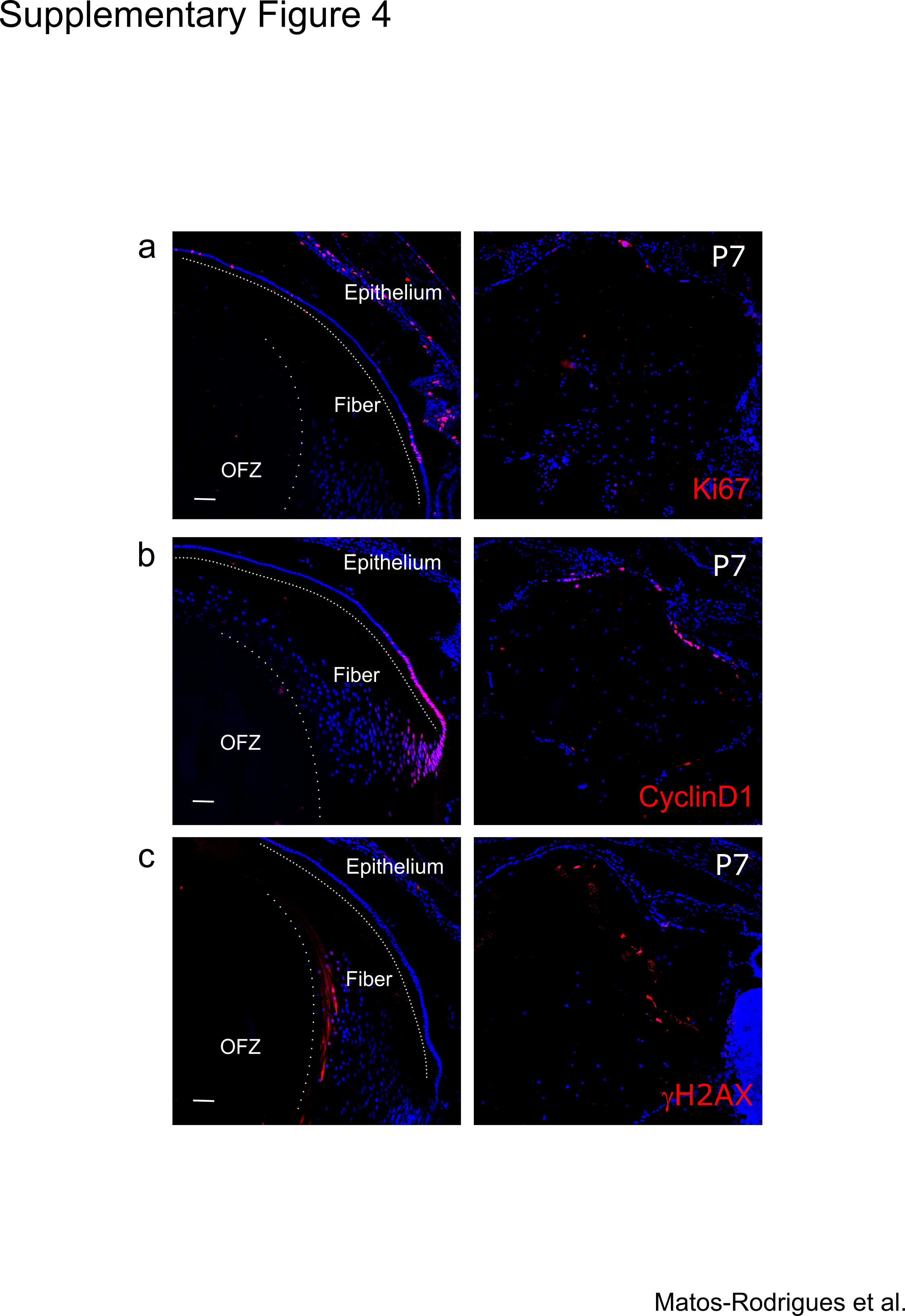

Supplement: Supplementary file 4 — SUPP FIG 4 [file 41419_2020_3090_MOESM4_ESM.png]

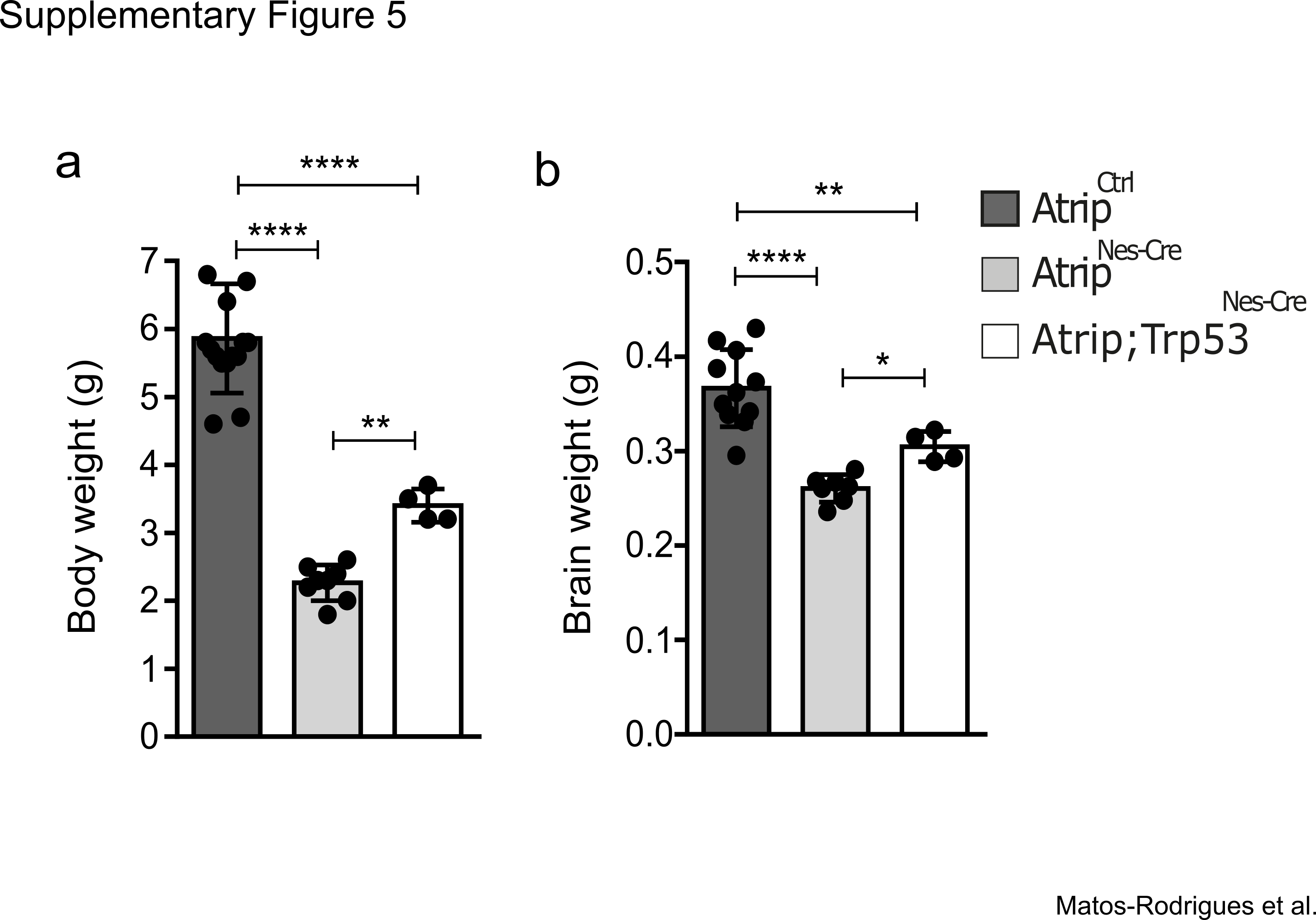

Supplement: Supplementary file 5 — SUPP FIG 5 [file 41419_2020_3090_MOESM5_ESM.png]
