# Supplementary material for: ATRIP protects progenitor cells against DNA damage in vivo
Source: Cell Death Dis. 2020 Oct 28;11(10):923. doi: 10.1038/s41419-020-03090-9 (PMC7591577; doi:10.1038/s41419-020-03090-9)
Supplement: Supplementary file 6 — Supp figure legends [file 41419_2020_3090_MOESM6_ESM.pdf]

Supplementary figure legends

Supplementary figure 1: *Atrip* is essential for brain development: (a) Representative images of H&E staining of P7 brain sections from *Atrip*^{Ctrl}, *Atrip*^{Het} and *Atrip*$^{Nes\text{-}Cre}$ mice of the body. The dotted line highlights the developmental defects observed in the cerebellum, cortex, hippocampus and striatum after *Atrip* inactivation. **(b)** Representative high-magnifications images of H&E stained sections of cerebellum, olfactory bulb and cortex from *Atrip*^{Ctrl}, *Atrip*^{Het} and *Atrip*$^{Nes\text{-}Cre}$ mice at P7.

Supplementary figure 2: *Atrip* and *Atr* expression pattern during lens development. (a, b) Real-time RT-PCR using specific primers for *Atr* and *Atrip* at 6 stages (E15.5, P0, P4, P9, P15. P60) of mouse lens development. *GPI-1* and *Actb* were used as internal control targets. The number of biological samples analyzed is represented as the dots in the graphs.

Supplementary figure 3: Inactivation of *Atrip* in the lens surface ectoderm severely impairs eye development (a) Representative images of P21 control *Atrip*^{Ctrl} and *Atrip*$^{Le\text{-}Cre}$ eyes**. (b)** Eye volume measurements revealed severe impairment of eye growth after ATRIP loss in the surface ectoderm. The number of biological samples analyzed is represented as the dots in the graphs. Error bars indicate SEM. *** $p<0.001$.

Supplementary figure 4: ATRIP loss severely compromise lens development. (a-c) Representative images of P7 lens sections from *Atrip*^{Ctrl} and *Atrip*$^{Nes\text{-}Cre}$ mice stained for Ki67 **(a)**, cyclin D1 **(b)** and γH2AX **(c)** at P7. Scale bar: 100 μm.

Supplementary figure 5: Inactivation of Trp53 partially rescues brain and weight following CNS-specific inactivation of *Atrip*. (a,b) Quantification of the body **(a)** and brain **(b)** weight in $\textit{Atrip}^{Ctrl}$, $\textit{Atrip}^{Nes\text{-}Cre}$ and cDKO ($\textit{Atrip; Trp53}^{Nes\text{-}Cre}$) mice at P7. The number of biological samples analyzed is represented as the dots in the graphs. Error bars indicate SEM ** $p<0.01$, *** $p<0.001$, **** $p<0.0001$.
